# On the Exact Variance of Tsallis Entanglement Entropy in a Random Pure State

**DOI:** 10.3390/e21050539

**Published:** 2019-05-27

**Authors:** Lu Wei

**Affiliations:** Department of Electrical and Computer Engineering, University of Michigan, Dearborn, MI 48128, USA; luwe@umich.edu

**Keywords:** entanglement entropy, quantum information theory, random matrix theory, variance

## Abstract

The Tsallis entropy is a useful one-parameter generalization to the standard von Neumann entropy in quantum information theory. In this work, we study the variance of the Tsallis entropy of bipartite quantum systems in a random pure state. The main result is an exact variance formula of the Tsallis entropy that involves finite sums of some terminating hypergeometric functions. In the special cases of quadratic entropy and small subsystem dimensions, the main result is further simplified to explicit variance expressions. As a byproduct, we find an independent proof of the recently proven variance formula of the von Neumann entropy based on the derived moment relation to the Tsallis entropy.

## 1. Introduction

Classical information theory is the theory behind the modern development of computing, communication, data compression, and other fields. As its classical counterpart, quantum information theory aims at understanding the theoretical underpinnings of quantum science that will enable future quantum technologies. One of the most fundamental features of quantum science is the phenomenon of quantum entanglement. Quantum states that are highly entangled contain more information about different parts of the composite system.

As a step to understand quantum entanglement, we choose to study the entanglement property of quantum bipartite systems. The quantum bipartite model, proposed in the seminal work of Page [1], is a standard model for describing the interaction of a physical object with its environment for various quantum systems. In particular, we wish to understand the degree of entanglement as measured by the entanglement entropies of such systems. The statistical behavior of entanglement entropies can be understood from their moments. In principle, the knowledge of all integer moments determines uniquely the distribution of the considered entropy as it is supported in a finite interval (cf. (5) below). This is also known as Hausdorff’s moment problem [2,3]. In practice, a finite number of moments can be utilized to construct approximations to the distribution of the entropy, where the higher moments describe the tail distribution that provides crucial information such as whether the mean entropy is a typical value [4]. Of particular importance is the second moment (variance) that governs the fluctuation of the entropy around the mean value. With the first two moments, one could already construct an upper bound to the probability of finding a state with entropy lower than the mean entropy by using the concentration of measure techniques [4].

The existing knowledge in the literature is mostly focused on the von Neumann entropy [1,4,5,6,7,8,9,10], where its first three exact moments are known. In this work, we consider the Tsallis entropy [11], which is a one-parameter generalization of the von Neumann entropy. The Tsallis entropy enjoys certain advantages in describing quantum entanglement. For example, it overcomes the inability of the von Neumann entropy to model systems with long-range interactions [12]. The Tsallis entropy also has the unique nonadditivity (also known as nonextensivity) property, whose physical relevance to quantum systems has been increasingly identified [13]. In the literature, the mean value of the Tsallis entropy was derived by Malacarne–Mendes–Lenzi [12]. The focus of this work is to study its variance.

The paper is organized as follows. In Section 2, we introduce the quantum bipartite model and the entanglement entropies. In Section 3, an exact variance formula of the Tsallis entropy in terms of finite sums of terminating hypergeometric functions is derived, which is the main result of this paper. As a byproduct, we provide in Appendix A another proof to the recently proven [4,10] Vivo–Pato–Oshanin’s conjecture [9] on the variance of the von Neumann entropy. In Section 4, the derived variance formula of the Tsallis entropy is further simplified to explicit expressions in the special cases of quadratic entropy and small subsystem dimensions. We summarize the main results and point out a possible approach to study the higher moments in Section 5.

## 2. Bipartite System and Entanglement Entropy

We consider a composite quantum system consisting of two subsystems *A* and *B* of Hilbert space dimensions *m* and *n*, respectively. The Hilbert space $H_{A+B}$ of the composite system is given by the tensor product of the Hilbert spaces of the subsystems, $H_{A+B}=H_{A}⊗H_{B}$. The random pure state (as opposed to the mixed state) of the composite system is written as a linear combination of the random coefficients $x_{i,j}$ and the complete basis $\left\{|i^{A}\rangle \right\}$ and $\left\{|j^{B}\rangle \right\}$ of $H_{A}$ and $H_{B}$, $|\psi \rangle =\sum_{i=1}^{m}\sum_{j=1}^{n}x_{i,j}|i^{A}\rangle ⊗|j^{B}\rangle$. The corresponding density matrix $\rho =|\psi \rangle \langle \psi |$ has the natural constraint tr(ρ)=1. This implies that the m×n random coefficient matrix $X=(x_{i,j})$ satisfies:

(1) $$\mathrm{tr}\left({\mathrm{XX}}^{†}\right)=1.$$

Without loss of generality, it is assumed that m≤n. The reduced density matrix $\rho_{A}$ of the smaller subsystem *A* admits the Schmidt decomposition $\rho_{A}=\sum_{i=1}^{m}\lambda_{i}|\phi_{i}^{A}\rangle \langle \phi_{i}^{A}|$, where $\lambda_{i}$ is the $i^{\mathrm{th}}$ largest eigenvalue of ${\mathrm{XX}}^{†}$. The conservation of probability (1) now implies the constraint $\sum_{i=1}^{m}\lambda_{i}=1$. The probability measure of the random coefficient matrix X is the Haar measure, where the entries are uniformly distributed over all the possible values satisfying the constraint (1). The resulting eigenvalue density of ${\mathrm{XX}}^{†}$ is (see, e.g., [1]),

(2) $$f\left(\lambda \right)=\frac{\Gamma (mn)}{c}\;\delta \left(1-\sum_{i=1}^{m}\lambda_{i}\right)\prod_{1\leq i<j\leq m}{\left(\lambda_{i}-\lambda_{j}\right)}^{2}\prod_{i=1}^{m}\lambda_{i}^{n-m},$$

where δ(·) is the Dirac delta function and the constant:

(3) $$c=\prod_{i=1}^{m}\Gamma \left(n-i+1\right)\Gamma \left(i\right).$$

The random matrix ensemble (2) is also known as the (unitary) fixed-trace ensemble. The above-described quantum bipartite model is useful in modeling various quantum systems. For example, in [1], the subsystem *A* is a black hole, and the subsystem *B* is the associated radiation field. In another example [14], the subsystem *A* is a set of spins, and the subsystem *B* represents the environment of a heat bath.

The degree of entanglement of quantum systems can be measured by the entanglement entropy, which is a function of the eigenvalues of ${\mathrm{XX}}^{†}$. The function should monotonically increase from the separable state ($\lambda_{1}=1$, $\lambda_{2}=\cdots =\lambda_{m}=0$) to the maximally-entangled state ($\lambda_{1}=\lambda_{2}=\cdots \lambda_{m}=1/m$). The most well-known entanglement entropy is the von Neumann entropy:

(4) $$S=-\sum_{i=1}^{m}\lambda_{i}\ln\lambda_{i},$$

which achieves the separable state and maximally-entangled state when S=0 and when S=lnm, respectively. A one-parameter generalization of the von Neumann entropy is the Tsallis entropy [11]:

(5) $$T=\frac{1}{q-1}\left(1-\sum_{i=1}^{m}\lambda_{i}^{q}\right),\;\;\;\;q\in R\backslash \left\{0\right\},$$

which, by l’Hôpital’s rule, reduces to the von Neumann entropy (4) when the non-zero real parameter *q* approaches one. The Tsallis entropy (5) achieves the separable state and maximally-entangled state when T=0 and $T=\left(m^{q-1}-1\right)/\left(q-1\right)m^{q-1}$, respectively. In some aspects, the Tsallis entropy provides a better description of the entanglement. For example, it overcomes the inability of the von Neumann entropy to model systems with long-range interactions [12]. The Tsallis entropy also has a definite concavity for any *q*, i.e., being convex for q<0 and concave for q>0. We also point out that by studying the moments of the Tsallis entropy (5) first, one may recover the moments of the von Neumann entropy (4) in a relatively simpler manner as opposed to directly working with the von Neumann entropy. The advantage of this indirect approach has been very recently demonstrated in the works [4,15]. In the same spirit, we will also provide in Appendix A another proof to the variance of the von Neumann entropy starting from the relation to the Tsallis entropy.

In the literature, the first moment of the von Neumann entropy $E_{f}\;\left[S\right]$ (the subscript *f* emphasizes that the expectation is taken over the fixed-trace ensemble (2)) was conjectured by Page [1]. Page’s conjecture was proven independently by Foong and Kanno [5], Sánchez-Ruiz [6], Sen [7], and Adachi–Toda–Kubotani [8]. Recently, an expression for the variance of the von Neumann entropy $V{\;}_{f}\;\left[S\right]$ was conjectured by Vivo–Pato–Oshanin (VPO) [9], which was subsequently proven by the author [10]. Bianchi and Donà [4] provided an independent proof to VPO’s conjecture very recently, where they also derived the third moment. For the Tsallis entropy, the first moment $E_{f}\;\left[T\right]$ was derived by Malacarne–Mendes–Lenzi [12]. The task of the present work is to study the variance of the Tsallis entropy $V{\;}_{f}\;\left[T\right]$.

## 3. Exact Variance of the Tsallis Entropy

Similar to the case of the von Neumann entropy [1,10], the starting point of the calculation is to convert the moments defined over the fixed-traced ensemble (2) to the well-studied Laguerre ensemble, whose correlation functions are explicitly known. Before discussing the moments conversion approach, we first set up necessary definitions relevant to the Laguerre ensemble. By construction (1), the random coefficient matrix X is naturally related to a Wishart matrix ${\mathrm{YY}}^{†}$ as:

(6) $${\mathrm{XX}}^{†}=\frac{{\mathrm{YY}}^{†}}{\mathrm{tr}\left({\mathrm{YY}}^{†}\right)},$$

where Y is an m×n (m≤n) matrix of independently and identically distributed complex Gaussian entries (complex Ginibre matrix). The density of the eigenvalues $0<\theta_{m}<\cdots <\theta_{1}<\infty$ of ${\mathrm{YY}}^{†}$ equals [16]:

(7) $$g\left(\theta \right)=\frac{1}{c}\prod_{1\leq i<j\leq m}{\left(\theta_{i}-\theta_{j}\right)}^{2}\prod_{i=1}^{m}\theta_{i}^{n-m}e^{-\theta_{i}},$$

where *c* is the same as in (3), and the above ensemble is known as the Laguerre ensemble. The trace of the Wishart matrix:

(8) $$r=\mathrm{tr}\left({\mathrm{YY}}^{†}\right)=\sum_{i=1}^{m}\theta_{i}$$

follows a gamma distribution with the density [9]:

(9) $$h_{mn}\left(r\right)=\frac{1}{\Gamma (mn)}e^{-r}r^{mn-1},\;\;\;\;r\in \left[0,\infty \right).$$

The relation (6) induces the change of variables:

(10) $$\lambda_{i}=\frac{\theta_{i}}{r},\;\;\;\;i=1,\ldots ,m,$$

that leads to a well-known relation (see, e.g., [1]) among the densities (2), (7), and (9) as:

(11) $$f\left(\lambda \right)h_{mn}\left(r\right)\;dr\prod_{i=1}^{m}\;d\lambda_{i}=g\left(\theta \right)\prod_{i=1}^{m}\;d\theta_{i}.$$

This implies that *r* is independent of each $\lambda_{i}$, i=1,…,m, since their densities factorize.

For the von Neumann entropy (4), the relation (11) has been exploited to convert the first two moments [1,10] from the fixed-trace ensemble (2) to the Laguerre ensemble (7). The moments conversion was an essential starting point in proving the conjectures of Page [1,6] and Vivo–Pato–Oshanin [10]. We now show that the moments conversion approach can be also applied to study the Tsallis entropy. We first define:

(12) $$L=\sum_{i=1}^{m}\theta_{i}^{q}$$

as the induced Tsallis entropy of the Laguerre ensemble (7). Here, for the convenience of the discussion, we have defined the induced entropy, which may not have the physical meaning of an entropy. Using the change of variables (10), the *k*th power of the Tsallis entropy (5) can be written as:

(13) Tk=1(q−1)k1−Lrqk=1(q−1)k∑i=0k(−1)ikiLirqi

and thus, we have:

(14) EfTk=1(q−1)k∑i=0k(−1)ikiEfLirqi.

The expectation on the left-hand side is computed as:

(15) $$\begin{array}{ccc} E_{f}\;\left[\frac{L^{i}}{r^{qi}}\right] & = & \int_{\lambda }\frac{L^{i}}{r^{qi}}f\left(\lambda \right)\prod_{i=1}^{m}\;d\lambda_{i} \end{array}$$

(16) $$\begin{array}{cc} = & \int_{\lambda }\frac{L^{i}}{r^{qi}}f\left(\lambda \right)\prod_{i=1}^{m}\;d\lambda_{i}\int_{r}h_{mn+qi}\left(r\right)\;dr \end{array}$$

(17) $$\begin{array}{cc} = & \frac{\Gamma (mn)}{\Gamma (mn+qi)}\int_{\lambda }\int_{r}L^{i}f\left(\lambda \right)h_{mn}\left(r\right)\;dr\prod_{i=1}^{m}\;d\lambda_{i} \end{array}$$

(18) $$\begin{array}{cc} = & \frac{\Gamma (mn)}{\Gamma (mn+qi)}E_{g}\;\left[L^{i}\right], \end{array}$$

where the multiplication of an appropriate constant $1=\int_{r}h_{mn+qi}\left(r\right)\;dr$ in (16) along with the fact that $r^{-qi}h_{mn+qi}\left(r\right)=\Gamma \left(mn\right)h_{mn}\left(r\right)/\Gamma \left(mn+qi\right)$ lead to (17), and the last equality (18) is established by the change of measures (11). Inserting (18) into (14), the *k*th moment of the Tsallis entropy (5) is written as a sum involving the first *k* moments of the induced Tsallis entropy (12) as:

(19) EfTk=Γ(mn)(q−1)k∑i=0kki(−1)iΓ(mn+qi)EgLi.

With the above relation (19), the computation of moments over the less tractable correlation functions of the fixed-trace ensemble (2) is now converted to the one over the Laguerre ensemble (7), which will be calculated explicitly. In particular, computing the variance $V{\;}_{f}\;\left[T\right]=E_{f}\;\left[T^{2}\right]-E_{f}^{2}\;\left[T\right]$ requires the moments relation (19) for k=1,

(20) $$E_{f}\;\left[T\right]=\frac{1}{q-1}\left(1-\frac{\Gamma (mn)}{\Gamma (mn+q)}E_{g}\;\left[L\right]\right)$$

and k=2,

(21) $$E_{f}\;\left[T^{2}\right]=\frac{1}{{\left(q-1\right)}^{2}}\left(1-\frac{2\Gamma (mn)}{\Gamma (mn+q)}E_{g}\;\left[L\right]+\frac{\Gamma (mn)}{\Gamma (mn+2q)}E_{g}\;\left[L^{2}\right]\right),$$

where the first moment relation (20) has also appeared in [12]. It is seen from (21) that the essential task now is to compute $E_{g}\;\left[L\right]$ and $E_{g}\;\left[L^{2}\right]$. Before proceeding to the calculation, we point out that in the limit q→1, the derived second moments relation (21) leads to a new proof to the recently proven variance formula of the von Neumann entropy [10] with details provided in the Appendix A.

The computation of $E_{g}\;\left[L\right]$ and $E_{g}\;\left[L^{2}\right]$ involves one and two arbitrary eigenvalue densities, denoted respectively by $g_{1}\left(x_{1}\right)$ and $g_{2}\left(x_{1},x_{2}\right)$, of the Laguerre ensemble as:

(22) $$\begin{array}{ccc} E_{g}\;\left[L\right] & = & m\int_{0}^{\infty }\;\;x_{1}^{q}\;g_{1}\left(x_{1}\right)\;dx_{1}, \end{array}$$

(23) $$\begin{array}{ccc} E_{g}\;\left[L^{2}\right] & = & m\int_{0}^{\infty }\;\;x_{1}^{2q}\;g_{1}\left(x_{1}\right)\;dx_{1}+m\left(m-1\right)\int_{0}^{\infty }\;\;\int_{0}^{\infty }\;\;x_{1}^{q}x_{2}^{q}\;g_{2}\left(x_{1},x_{2}\right)\;dx_{1}\;dx_{2}. \end{array}$$

In general, the joint density of *N* arbitrary eigenvalues $g_{N}\left(x_{1},\cdots ,x_{N}\right)$ is related to the *N*-point correlation function:

(24) $$X_{N}\left(x_{1},\cdots ,x_{N}\right)=\det{\left(K\left(x_{i},x_{j}\right)\right)}_{i,j=1}^{N}$$

as [16] $g_{N}\left(x_{1},\cdots ,x_{N}\right)=X_{N}\left(x_{1},\cdots ,x_{N}\right)\left(m-N\right)!/m!$, where det(·) is the matrix determinant and the symmetric function $K(x_{i},x_{j})$ is the correlation kernel. In particular, we have:

(25) $$\begin{array}{ccc} g_{1}\left(x_{1}\right) & = & \frac{1}{m}K\left(x_{1},x_{1}\right), \end{array}$$

(26) $$\begin{array}{ccc} g_{2}\left(x_{1},x_{2}\right) & = & \frac{1}{m(m-1)}\left(K\left(x_{1},x_{1}\right)K\left(x_{2},x_{2}\right)-K^{2}\left(x_{1},x_{2}\right)\right), \end{array}$$

and the correlation kernel $K(x_{i},x_{j})$ of the Laguerre ensemble can be explicitly written as [16]:

(27) $$K\left(x_{i},x_{j}\right)=\sqrt{e^{-x_{i}-x_{j}}{\left(x_{i}x_{j}\right)}^{n-m}}\sum_{k=0}^{m-1}\frac{C_{k}\left(x_{i}\right)C_{k}\left(x_{j}\right)}{k!(n-m+k)!},$$

where:

(28) $$C_{k}\left(x\right)={\left(-1\right)}^{k}k!L_{k}^{\left(n-m\right)}\left(x\right)$$

with:

(29) Lk(n−m)(x)=∑i=0k(−1)in−m+kk−ixii!

the (generalized) Laguerre polynomial being of degree *k*. The Laguerre polynomials satisfy the orthogonality relation [16]:

(30) $$\int_{0}^{\infty }\;\;x^{n-m}e^{-x}L_{k}^{\left(n-m\right)}\left(x\right)L_{l}^{\left(n-m\right)}\left(x\right)\;dx=\frac{(n-m+k)!}{k!}\delta_{kl},$$

where $\delta_{kl}$ is the Kronecker delta function. It is known that the one-point correlation function admits a more convenient representation as [6,16]:

(31) $$X_{1}\left(x\right)=K\left(x,x\right)=\frac{m!}{(n-1)!}x^{n-m}e^{-x}\left({\left(L_{m-1}^{\left(n-m+1\right)}\left(x\right)\right)}^{2}-L_{m-2}^{\left(n-m+1\right)}\left(x\right)L_{m}^{\left(n-m+1\right)}\left(x\right)\right).$$

We also need the following integral identity, due to Schrödinger [17], that generalizes the integral (30) to:

(32) As,t(α,β)(q)=∫0∞xqe−xLs(α)(x)Lt(β)(x)dx=(−1)s+t∑k=0min(s,t)q−αs−kq−βt−kΓ(k+q+1)k!,q>−1.

With the above preparation, we now proceed to the calculation of $E_{g}\;\left[L\right]$ and $E_{g}\;\left[L^{2}\right]$. Inserting (25) and (31) into (22) and defining further:

(33) $$A_{s,t}=A_{s,t}^{\left(n-m+1,n-m+1\right)}\left(n-m+q\right),$$

one obtains by using (32) that:

(34) EgL=m!(n−1)!Am−1,m−1−Am−2,m=m!(n−1)!(∑k=0m−1q−1m−k−12Γ(n−m+q+k+1)k!−∑k=0m−2q−1m−k−2q−1m−kΓ(n−m+q+k+1)k!),

which is valid for q>−1. The first moment expression in the above form has been obtained in [12], and we continue to show that it can be compactly written as a terminating hypergeometric function of the unit argument. Indeed, since:

(35) q−1−1q−11Γ(n+q)(m−1)!=0,q>−1\{0},

we have:

(36) EgL=m!(n−1)!∑k=0m−1Γ(n−m+q+k+1)k!q−1m−k−12−q−1m−k−2q−1m−k=m!Γ2(q)(n−1)!∑k=0m−1Γ(n+q−k)(m−k−1)!1k!2Γ2(q−k)−1(k−1)!(k+1)!Γ(q−k+1)Γ(q−k−1)

(37) $$\begin{array}{cc} = & \frac{m!\Gamma (q+1)\Gamma (q)}{(n-1)!}\sum_{k=0}^{m-1}\frac{\Gamma (n+q-k)}{(m-k-1)!\Gamma (q-k+1)\Gamma (q-k)k!(k+1)!} \end{array}$$

(38) $$\begin{array}{cc} = & \frac{m!\Gamma (q+1)\Gamma (q)}{(n-1)!}\frac{\Gamma (n+q)}{\left(m-1)!\Gamma (q+1)\Gamma (q\right)}\sum_{k=0}^{m-1}\frac{{\left(1-m\right)}_{k}{\left(-q\right)}_{k}{\left(1-q\right)}_{k}}{{\left(1-n-q\right)}_{k}{\left(2\right)}_{k}}\frac{1}{k!} \end{array}$$

(39) =mΓ(n+q)(n−1)!3F21−m,−q,1−q1−n−q,2;1,q>−1\{0},

where the second equality follows from the change of variable k→m−1−k, and (38) is obtained by repeated use of the identity:

(40) $$\Gamma \left(m-k\right)=\frac{{\left(-1\right)}^{k}}{{\left(1-m\right)}_{k}}\Gamma \left(m\right)$$

with ${\left(a\right)}_{n}=\Gamma \left(a+n\right)/\Gamma \left(a\right)$ being Pochhammer’s symbol; and (39) is obtained by the series definition of the hypergeometric function:

(41) pFqa1⋯apb1⋯bq;z=∑k=0∞(a1)k⋯(ap)k(b1)k⋯(bq)kzkk!

that reduces to a finite sum if one of the parameters $a_{i}$ is a negative integer. Inserting (39) into (20), we arrive at a compact expression for the first moment of the Tsallis entropy as:

(42) EfT=1q−11−m(mn−1)!Γ(n+q)(n−1)!Γ(mn+q)3F21−m,−q,1−q1−n−q,2;1,q>−1\{0}.

We now calculate $E_{g}\;\left[L^{2}\right]$. Inserting (25) and (26) into (23), one has:

(43) EgL2=I1−I2+m2Γ2(n+q)(n−1)!23F21−m,−q,1−q1−n−q,2;12,

where:

(44) $$\begin{array}{ccc} I_{1} & = & \int_{0}^{\infty }\;\;x_{1}^{2q}\;K\left(x_{1},x_{1}\right)\;dx_{1}, \end{array}$$

(45) $$\begin{array}{ccc} I_{2} & = & \int_{0}^{\infty }\;\;\int_{0}^{\infty }\;\;x_{1}^{q}x_{2}^{q}\;K^{2}\left(x_{1},x_{2}\right)\;dx_{1}\;dx_{2}, \end{array}$$

and we have used the result (39) with the fact that:

(46) $$\int_{0}^{\infty }\;\;x^{q}K\left(x,x\right)\;dx=E_{g}\;\left[L\right].$$

The integral $I_{1}$ can be read off from the steps that led to (39) by replacing *q* with 2q as:

(47) I1=mΓ(n+2q)(n−1)!3F21−m,−2q,1−2q1−n−2q,2;1.

Inserting (27) into (45) and defining further (cf. (32)):

(48) $$A_{s,t}=A_{s,t}^{\left(n-m,n-m\right)}\left(n-m+q\right),$$

the integral $I_{2}$ is written as:

(49) $$I_{2}=\sum_{k=0}^{m-1}\frac{k{!}^{2}A_{k,k}^{2}}{\left(n-m+k\right){!}^{2}}+2\sum_{j=1}^{m-1}\sum_{i=0}^{j-1}\frac{i!j!A_{i,j}^{2}}{(n-m+i)!(n-m+j)!},$$

where by using (32) and (40), we obtain:

(50) $$\begin{array}{ccc} A_{i,j} & = & \Gamma^{2}\left(q+1\right)\sum_{k=0}^{i}\frac{\Gamma (n-m+q+k+1)}{\Gamma (q-i+k+1)\Gamma (q-j+k+1)(i-k)!(j-k)!k!} \end{array}$$

(51) $$\begin{array}{cc} = & \frac{\Gamma (n-m+q+1)}{\Gamma (q-i+1)\Gamma (q-j+1)i!j!}\sum_{k=0}^{i}\frac{{\left(-i\right)}_{k}{\left(-j\right)}_{k}{\left(n-m+q+1\right)}_{k}}{{\left(q-i+1\right)}_{k}{\left(q-j+1\right)}_{k}}\frac{1}{k!} \end{array}$$

(52) =Γ(n−m+q+1)Γ(q−i+1)Γ(q−j+1)i!j!3F2−i,−j,n−m+q+1q−i+1,q−j+1;1,

and similarly:

(53) Ak,k=Γ(n−m+q+1)Γ2(q−k+1)k!23F2−k,−k,n−m+q+1q−k+1,q−k+1;1.

Finally, by inserting (47), (49), (52), and (53) into (43), we arrive at:

(54) EgL2=m2Γ2(n+q)(n−1)!23F21−m,−q,1−q1−n−q,2;12+mΓ(n+2q)(n−1)!3F21−m,−2q,1−2q1−n−2q,2;1−Γ4(q+1)Γ2(n−m+q+1)∑i=0m−1L2(i,i)+2∑j=1m−1∑i=0j−1L2(i,j),q>−1\{0},

where the symmetric function L(i,j)=L(j,i) is:

(55) L(i,j)=3F2−i,−j,n−m+q+1q−i+1,q−j+1;1Γ(q−i+1)Γ(q−j+1)i!j!(n−m+i)!(n−m+j)!.

With the derived first two moments (39) and (54) and the relations (20) and (21), an exact variance formula of the Tsallis entropy is obtained.

## 4. Special Cases

Though the derived results (39) and (54) may not be further simplified for an arbitrary *m*, *n*, and *q*, we will show that explicit variance expressions can be obtained in some special cases of practical relevance.

### 4.1. Quadratic Entropy q=2

In the special case q=2, the Tsallis entropy (5) reduces to the quadratic entropy:

(56) $$T=1-\sum_{i=1}^{m}\lambda_{i}^{2},$$

which was first considered in physics by Fermi [12]. The quadratic entropy (56) is the only entropy among all possible *q* values that satisfies the information invariance and continuity criterion [18].

By the series representations (38) and (51), the first two moments in the case q=2 are directly computed as:

(57) $$\begin{array}{ccc} E_{g}\;\left[L\right] & = & mn(m+n), \end{array}$$

(58) $$\begin{array}{ccc} E_{g}\;\left[L^{2}\right] & = & mn\left(mn^{3}+2m^{2}n^{2}+4n^{2}+m^{3}n+10mn+4m^{2}+2\right). \end{array}$$

By (20) and (21), we immediately have:

(59) $$\begin{array}{lll} \;\;\;\;\;\;\;E_{f}\;\left[T\right] & = & \frac{mn-m-n+1}{mn+1}, \end{array}$$

(60) $$\begin{array}{lll} \;\;\;\;\;\;\;E_{f}\;\left[T^{2}\right] & = & \frac{\left(m-1)(n-1\right)}{\left(mn+1)(mn+2)(mn+3\right)}\left(m^{2}n^{2}-mn^{2}-m^{2}n+5mn-4n-4m+8\right), \end{array}$$

which lead to the variance of Tsallis entropy for q=2 as:

(61) $$V{\;}_{f}\;\left[T\right]=\frac{2\left(m^{2}-1\right)\left(n^{2}-1\right)}{{\left(mn+1\right)}^{2}\left(mn+2\right)\left(mn+3\right)}.$$

Finally, we note that explicit variance expressions for other positive integer values of *q* can be similarly obtained.

### 4.2. Subsystems of Dimensions m = 2 and m = 3

We now consider the cases when dimensions *m* of the smaller subsystems are small. This is a relevant scenario for subsystems consisting of, for example, only a few entangled particles [14]. For m=2 with any *n* and *q*, the series representations (38) and (51) directly lead to the results:

(62) $$\begin{array}{ccc} E_{g}\;\left[L\right] & = & \frac{q^{2}+q+2n-2}{(n-1)!}\Gamma \left(q+n-1\right), \end{array}$$

(63) $$\begin{array}{ccc} E_{g}\;\left[L^{2}\right] & = & \frac{2}{(n-1)!}\left(\frac{\Gamma (q+n-1)\Gamma (q+n)}{(n-2)!}+\left(2q^{2}+q+n-1\right)\Gamma \left(2q+n-1\right)\right). \end{array}$$

In the same manner, for m=3 with any *n* and *q*, we obtain:

(64) $$\begin{array}{ccc} E_{g}\;\left[L\right] & = & \frac{6n\left(q^{2}+q-3\right)+\left(q-2\right)\left(q-1\right)\left(q+2\right)\left(q+3\right)+6n^{2}}{2(n-1)!}\Gamma \left(q+n-2\right), \end{array}$$

(65) $$\begin{array}{ccc} E_{g}\;\left[L^{2}\right] & = & \frac{6n\left(q^{2}+q-3\right)+q^{4}+4q^{3}-7q^{2}-10q+12+6n^{2}}{(n-1)!(n-2)!}\Gamma \left(q+n-2\right)\Gamma \left(q+n-1\right)+\\ & & \frac{3n\left(4q^{2}+2q-3\right)+8q^{4}+8q^{3}-14q^{2}-8q+6+3n^{2}}{(n-1)!}\Gamma \left(2q+n-2\right). \end{array}$$

The corresponding variances are obtained by keeping in mind the relations (20) and (21). For m≥4, explicit variance expressions can be similarly calculated. However, it does not seem promising to find an explicit variance formula valid for any *m*, *n*, and *q*.

## 5. Summary and Perspectives on Higher Moments

We studied the exact variance of the Tsallis entropy, which is a one-parameter (*q*) generalization of the von Neumann entropy. The main result is an exact variance expression (54) valid for q>−1 as finite sums of terminating hypergeometric functions. For q=1, we find a short proof to the variance formula of the degenerate case of the von Neumann entropy in the Appendix. For other special cases of the practical importance of q=2, m=2, and m=3, explicit variance expressions have been obtained in (61), (63), and (65), respectively.

We end this paper with some perspectives on the higher moments of the Tsallis entropy. In principle, the higher moments can be calculated by integrating over the correlation kernel (27) as demonstrated for the first two moments. In practice, the calculation becomes progressively complicated as the order of moments increases. Here, we outline an alternative path that may systematically lead to the moments of any order in a recursive manner.

We focus on the induced Tsallis entropy *L* as defined in (12) since the moments conversion is available (19). The starting point is the generating function of *L*:

(66) $$\begin{array}{ccc} \tau_{m}\left(t,q\right)=E_{g}\;\left[e^{tL}\right] & = & \frac{1}{c}\int_{0}^{\infty }\;\;\cdots \;\;\int_{0}^{\infty }\;\prod_{1\leq i<j\leq m}{\left(\theta_{i}-\theta_{j}\right)}^{2}\prod_{i=1}^{m}\theta_{i}^{n-m}e^{-\theta_{i}+t\theta_{i}^{q}}\;d\theta_{i} \end{array}$$

(67) $$\begin{array}{cc} = & \frac{1}{c}\det{\left(\int_{0}^{\infty }x^{i+j+n-m-2}e^{-x+tx^{q}}\;dx\right)}_{i,j=1}^{m}, \end{array}$$

which is a two-parameter (*t* and *q*) deformation of the Laguerre ensemble (7). Compared to the weight function $w\left(x\right)=x^{n-m}e^{-x}$ of the Laguerre ensemble, the deformation induces a new weight function:

(68) $$w\left(x\right)=x^{n-m}e^{-x+tx^{q}},$$

which generalizes the Toda deformation [19] $w\left(x\right)=x^{n-m}e^{-x+tx}$ with the parameter *q*. The basic idea to produce the moments systematically is to find some differential and difference equations of the generating function $\tau_{m}\left(t,q\right)$. The theory of integrable systems [16] may provide the possibility to obtain differential equations for the Hankel determinant (67) with respect to continuous variables *t* and *q*, as well as difference equations with respect to the discrete variable *m*. In particular, when *q* is a positive integer, the deformation (68) is known as multi-time Toda deformation [19], where much of the integrable structure is known [19].

## Appendix A. A New Proof to the Variance Formula of the von Neumann Entropy

Vivo, Pato, and Oshanin recently conjectured that the variance of the von Neumann entropy (4) in a random pure state (2) is [9]:

(A1) $$V{\;}_{f}\;\left[S\right]=-\psi_{1}\left(mn+1\right)+\frac{m+n}{mn+1}\psi_{1}\left(n\right)-\frac{\left(m+1)(m+2n+1\right)}{4n^{2}\left(mn+1\right)},$$

where:

(A2) $$\psi_{1}\left(x\right)=\frac{\;d^{2}\ln\Gamma \left(x\right)}{\;dx^{2}}$$

is the trigamma function. The conjecture was proven in [4,10], and here, we provide another proof starting from the relation (21),

(A3) $$E_{f}\;\left[T^{2}\right]=\frac{1}{{\left(q-1\right)}^{2}}\left(1-\frac{2\Gamma (mn)}{\Gamma (mn+q)}E_{g}\;\left[L\right]+\frac{\Gamma (mn)}{\Gamma (mn+2q)}E_{g}\;\left[L^{2}\right]\right).$$

To resolve the indeterminacy in the limit q→1, we apply twice l’Hôpital’s rule on both sides of the above equation:

(A4) $$E_{f}\;\left[S^{2}\right]=\lim_{q\to 1}E_{f}\;\left[T^{2}\right]=\frac{\Gamma (mn)}{2}{\left(-\frac{2}{\Gamma (mn+q)}E_{g}\;\left[L\right]+\frac{1}{\Gamma (mn+2q)}E_{g}\;\left[L^{2}\right]\right)}^{\prime \prime }{|}_{q=1},$$

where $f^{\prime }=\;df/\;dq$. Define an induced von Neumann entropy of the Laguerre ensemble (7):

(A5) $$R_{a}=\sum_{i=1}^{m}\theta_{i}\ln^{a}\theta_{i}$$

with $R_{1}$ further denoted by $R=R_{1}$; the right-hand side of (A4) can be evaluated by using the following facts:

(A6) $$\begin{array}{ccc} E_{g}\;\left[L\right]{|}_{q=1} & = & E_{r}\;\left[r\right], \end{array}$$

(A7) $$\begin{array}{ccc} E_{g}\;\left[L^{2}\right]{|}_{q=1} & = & E_{r}\;\left[r^{2}\right], \end{array}$$

(A8) $$\begin{array}{ccc} E_{g}{\;}^{\prime }\left[L\right]{|}_{q=1} & = & E_{g}\;\left[R\right], \end{array}$$

(A9) $$\begin{array}{ccc} E_{g}{\;}^{\prime \prime }\left[L\right]{|}_{q=1} & = & E_{g}\;\left[R_{2}\right], \end{array}$$

(A10) $$\begin{array}{ccc} E_{g}{\;}^{\prime }\left[L^{2}\right]{|}_{q=1} & = & 2E_{g}\;\left[rR\right], \end{array}$$

(A11) $$\begin{array}{ccc} E_{g}{\;}^{\prime \prime }\left[L^{2}\right]{|}_{q=1} & = & 2E_{g}\;\left[R^{2}\right]+2E_{g}\;\left[rR_{2}\right], \end{array}$$

and the definitions of the digamma function $\psi_{0}\left(x\right)=\;d\ln\Gamma \left(x\right)/\;dx$ and the trigamma function (A2) that give:

(A12) $$\begin{array}{ccc} \Gamma^{\prime }\left(q\right) & = & \Gamma \left(q\right)\psi_{0}\left(q\right), \end{array}$$

(A13) $$\begin{array}{ccc} \Gamma^{\prime \prime }\left(q\right) & = & \Gamma \left(q\right)\left(\psi_{1}\left(q\right)+\psi_{0}^{2}\left(q\right)\right), \end{array}$$

as:

(A14) $$\begin{array}{ccc} E_{f}\;\left[S^{2}\right] & = & \frac{1}{mn}\left(2E_{g}\;\left[R\right]\psi_{0}\left(mn+1\right)+E_{r}\;\left[r\right]\left(\psi_{1}\left(mn+1\right)-\psi_{0}^{2}\left(mn+1\right)\right)-E_{g}\;\left[R_{2}\right]\right)+\\ & & \frac{1}{mn(mn+1)}(E_{g}\;\left[R^{2}\right]+E_{g}\;\left[rR_{2}\right]-4E_{g}\;\left[rR\right]\psi_{0}\left(mn+2\right)-\\ & & 2E_{r}\;\left[r^{2}\right]\left(\psi_{1}\left(mn+2\right)-\psi_{0}^{2}\left(mn+2\right)\right)\;). \end{array}$$

In (A14), the first two moments of *r* are given by:

(A15) $$E_{r}\;\left[r\right]=mn,\;\;\;\;E_{r}\;\left[r^{2}\right]=mn\left(mn+1\right),$$

which are obtained from the $k^{\mathrm{th}}$ moment expression (cf. (9)):

(A16) $$E_{r}\;\left[r^{k}\right]=\frac{\Gamma (mn+k)}{\Gamma (mn)}.$$

The first two moments of the induced von Neumann entropy over the Laguerre ensemble $E_{g}\;\left[R\right]$ and $E_{g}\;\left[R^{2}\right]$ in (A14) have been computed in [6] and [7] as:

(A17) $$E_{g}\;\left[R\right]=mn\psi_{0}\left(n\right)+\frac{1}{2}m\left(m+1\right)$$

and in [10] as:

(A18) $$\begin{array}{ccc} E_{g}\;\left[R^{2}\right] & = & mn\left(m+n\right)\psi_{1}\left(n\right)+mn\left(mn+1\right)\psi_{0}^{2}\left(n\right)+m\left(m^{2}n+mn+m+2n+1\right)\psi_{0}\left(n\right)+\\ & & \frac{1}{4}m\left(m+1\right)\left(m^{2}+m+2\right), \end{array}$$

respectively. The remaining task is to calculate $E_{g}\;\left[rR\right]$, $E_{g}\;\left[R_{2}\right]$, and $E_{g}\;\left[rR_{2}\right]$ in (A14). This relies on the repeated use of the change of variables (10) and measures (11), which exploit the independence between *r* and λ. Indeed, we have:

(A19) $$\begin{array}{ccc} E_{g}\;\left[rR\right] & = & E_{g}\;\left[r\sum_{i=1}^{m}r\lambda_{i}\ln\left(r\lambda_{i}\right)\right] \end{array}$$

(A20) $$\begin{array}{cc} = & E_{r}\;\left[r^{2}\ln r\right]-E_{g}\;\left[r^{2}S\right] \end{array}$$

(A21) $$\begin{array}{cc} = & \frac{\Gamma (mn+2)}{\Gamma (mn)}\psi_{0}\left(mn+2\right)-E_{r}\;\left[r^{2}\right]E_{f}\;\left[S\right] \end{array}$$

(A22) $$\begin{array}{cc} = & mn\left(mn+1\right)\left(\psi_{0}\left(n\right)+\frac{1}{mn+1}+\frac{m+1}{2n}\right), \end{array}$$

where (A21) is obtained by (11) and the identity:

(A23) ∫0∞e−rra−1lnrdr=Γ(a)ψ0(a),Re(a)>0,

and (A22) is obtained by (A16) and the mean formula of the von Neumann entropy [1,5,6,7,8]:

(A24) $$E_{f}\;\left[S\right]=\psi_{0}\left(mn+1\right)-\psi_{0}\left(n\right)-\frac{m+1}{2n}.$$

Define a generalized von Neumann entropy to (4) as:

(A25) $$S_{a}=-\sum_{i=1}^{m}\lambda_{i}\ln^{a}\lambda_{i}$$

with $S=S_{1}$, we similarly have:

(A26) $$\begin{array}{ccc} E_{g}\;\left[rR_{2}\right] & = & E_{r}\;\left[r^{2}\ln^{2}r\right]-2E_{g}\;\left[r^{2}\ln rS\right]-E_{g}\;\left[r^{2}S_{2}\right] \end{array}$$

(A27) $$\begin{array}{cc} = & E_{r}\;\left[r^{2}\ln^{2}r\right]-2E_{r}\;\left[r^{2}\ln r\right]E_{f}\;\left[S\right]-E_{r}\;\left[r^{2}\right]E_{f}\;\left[S_{2}\right], \end{array}$$

where the term:

(A28) $$E_{r}\;\left[r^{2}\ln^{2}r\right]=mn\left(mn+1\right)\left(\psi_{1}\left(mn+2\right)+\psi^{2}\left(mn+2\right)\right)$$

is obtained by the identity:

(A29) ∫0∞e−rra−1ln2rdr=Γ(a)ψ1(a)+ψ02(a),Re(a)>0,

and it remains to calculate the term $E_{f}\;\left[S_{2}\right]$ in (A27),

(A30) $$\begin{array}{ccc} \;\;\;\;\;\;\;\;\;\;\;\;\;\;\;\;E_{f}\;\left[S_{2}\right] & = & \int_{\lambda }\left(-\frac{R_{2}}{r}-2S\ln r+\ln^{2}r\right)f\left(\lambda \right)\prod_{i=1}^{m}\;d\lambda_{i}\int_{r}h_{mn+1}\left(r\right)\;dr \end{array}$$

(A31) $$\begin{array}{cc} = & -\frac{1}{mn}E_{g}\;\left[R_{2}\right]-2\psi_{0}\left(mn+1\right)E_{f}\;\left[S\right]+\psi_{1}\left(mn+1\right)+\psi_{0}^{2}\left(mn+1\right) \end{array}$$

(A32) $$\begin{array}{cc} = & -\frac{1}{mn}E_{g}\;\left[R_{2}\right]+\psi_{1}\left(mn+1\right)-\psi_{0}^{2}\left(mn+1\right)+2\psi_{0}\left(mn+1\right)\left(\;\psi_{0}\left(n\right)+\frac{m+1}{2n}\;\right). \end{array}$$

It is seen that the term involving $E_{g}\;\left[R_{2}\right]$ in the above cancels the one in (A14). Finally, inserting (A15), (A17), (A18), (A22), (A27), and (A32) into (A14) and keeping in mind the mean formula (A24), we prove the variance formula (A1) after some necessary simplification by the identities:

(A33) $$\psi_{0}\left(l+n\right)=\psi_{0}\left(l\right)+\sum_{k=0}^{n-1}\frac{1}{l+k},\;\;\;\;\psi_{1}\left(l+n\right)=\psi_{1}\left(l\right)-\sum_{k=0}^{n-1}\frac{1}{{\left(l+k\right)}^{2}}.$$
